# Supplementary material for: Effect of Natural Disaster-Related Prenatal Maternal Stress on Child Development and Health: A Meta-Analytic Review
Source: Int J Environ Res Public Health. 2021 Aug 6;18(16):8332. doi: 10.3390/ijerph18168332 (PMC8391830; doi:10.3390/ijerph18168332)
Supplement: Supplementary file 1 [file ijerph-18-08332-s001.zip › Supplemental material_Search strategies.pdf]

Database	All Ovid MEDLINE(R) 1946 to Present
Line	Strategy
1	Exp Natural Disasters/ OR exp Cold Temperature/ OR exp Hot Temperature/ OR Volcanic Eruptions/ OR Snow/
2	(natural disaster* OR natural catastrophe* OR natural hazard* OR avalanche* OR landslide* OR mudslide* OR rockslide* OR earthquake* OR sinkhole* OR volcanic eruption* OR volcano* OR flood* OR tsunami* OR tidal wave* OR ocean tide* OR earth tide* OR hurricane* OR typhoon* OR limnic eruption* OR cyclone* OR cyclonic storm* OR blizzard* OR ice storm* OR tropical storm* OR hailstorm* OR hail storm* OR cold wave* OR heat wave* OR drought* OR thunderstorm* OR tornado* OR wildfire* OR forest fire* OR wild fire* OR bush fire* OR bushfire* OR brush fire* OR brushfire* OR peat fire* OR vegetation fire*).ti,ab.
3	1 OR 2
4	Embryonic structures/ OR Fetus/ OR Pregnant Women/ OR exp Pregnancy/ OR Maternal Exposure/ OR "Prenatal Exposure Delayed Effects"/
5	(embryo* OR fetus* OR foetus* OR fetal* OR foetal* OR transplacenta* OR pregnan* OR trans-placenta* OR utero* OR intrauterine OR intra-uterine OR antenatal* OR ante-natal* OR antepartum OR ante-partum OR prenatal* OR pre-natal* OR intrapartum OR intra-partum OR perinatal* OR peri-natal* OR neonatal* OR neo-natal*).ti,ab.
6	4 OR 5
7	exp infant/ OR exp child/ OR adolescent/
8	(baby OR babies OR neonate* OR neo-nate* OR newborn* OR new-born* OR infant* OR child* OR boy* OR girl* OR kid* OR pubescent* OR prepubescent* OR preteen* OR progen* OR juvenile* OR toddler* OR youth* OR offspring OR youngster* OR adolescen* OR teen*).ti,ab.
9	7 OR 8
10	3 AND 6 AND 9
11	limit 10 to ((chinese or english or french) and humans)

Database	Embase 1974 to 2019
Line	Strategy
1	Natural Disaster/ or avalanche/ or hurricane/ or drought/ or earthquake/ or flooding/ or landslide/ or tsunami/ or tornado/ or exp wildfire/
2	(natural disaster* OR natural catastrophe* OR natural hazard* OR avalanche* OR landslide* OR mudslide* OR rockslide* OR earthquake* OR sinkhole* OR volcanic eruption* OR volcano* OR flood* OR tsunami* OR tidal wave* OR ocean tide* OR earth tide* OR hurricane* OR typhoon* OR limnic eruption* OR cyclone* OR cyclonic storm* OR blizzard* OR ice storm* OR tropical storm* OR hailstorm* OR hail storm* OR cold wave* OR heat wave* OR drought* OR thunderstorm* OR tornado* OR wildfire* OR forest fire* OR wild fire* OR bush fire* OR bushfire* OR brush fire* OR brushfire* OR peat fire* OR vegetation fire*).ti,ab.
3	1 OR 2
4	Embryo/ OR Fetus/ OR Pregnant Woman/ OR exp Pregnancy/ OR Maternal Exposure/ OR Prenatal Exposure/
5	(embryo* OR fetus* OR foetus* OR fetal* OR foetal* OR transplacenta* OR pregnan* OR trans-placenta* OR utero* OR intrauterine OR intra-uterine OR antenatal* OR ante-natal* OR antepartum OR ante-partum OR prenatal* OR pre-natal* OR intrapartum OR intra-partum OR perinatal* OR peri-natal* OR neonatal* OR neo-natal*).ti,ab.
6	4 OR 5
7	exp child/ OR adolescent/ OR exp adolescence/ OR exp childhood/
8	(baby OR babies OR neonate* OR neo-nate* OR newborn* OR new-born* OR infant* OR child* OR boy* OR girl* OR kid* OR pubescent* OR prepubescent* OR preteen* OR progen* OR juvenile* OR toddler* OR youth* OR offspring OR youngster* OR adolescen* OR teen*).ti,ab.
9	7 OR 8
10	3 AND 6 AND 9
11	limit 10 to ((chinese or english or french) and humans)

Database	Web of Science<br>Indexes=SCI-EXPANDED, SSCI, A&HCI, CPCI-S, CPCI-SSH, ESCI<br>Timespan=All years
Line	Strategy
1	TS=("natural disaster*" OR "natural catastrophe*" OR "natural hazard*" OR avalanche* OR landslide* OR mudslide* OR rockslide* OR earthquake* OR sinkhole* OR "volcanic eruption*" OR volcano* OR flood* OR tsunami* OR "tidal wave*" OR "ocean tide*" OR "earth tide*" OR hurricane* OR typhoon* OR "limnic eruption*" OR cyclone* OR "cyclonic storm*" OR blizzard* OR "ice storm*" OR "tropical storm*" OR hailstorm* OR "hail storm*" OR "cold wave*" OR "heat wave*" OR drought* OR thunderstorm* OR tornado* OR wildfire* OR "forest fire*" OR "wild fire*" OR "bush fire*" OR bushfire* OR "brush fire*" OR brushfire* OR "peat fire*" OR "vegetation fire*")
2	TS=(embryo* OR fetus* OR foetus* OR fetal* OR foetal* OR transplacenta* OR pregnan* OR trans-placenta* OR utero* OR intrauterine OR intra-uterine OR antenatal* OR ante-natal* OR antepartum OR ante-partum OR prenatal* OR pre-natal* OR intrapartum OR intra-partum OR perinatal* OR peri-natal* OR neonatal* OR neo-natal*)
3	TS=(baby OR babies OR neonate* OR neo-nate* OR newborn* OR new-born* OR infant* OR child* OR boy* OR girl* OR kid* OR pubescent* OR prepubescent* OR preteen* OR progen* OR juvenile* OR toddler* OR youth* OR offspring OR youngster* OR adolescen* OR teen*)
4	#3 AND #2 AND #1
5	(#4) AND LANGUAGE: (English OR Chinese OR French)

Database	CINAHL Plus with Full Text
Line	Strategy
S1	(MH "Natural Disasters") OR (MH "Temperature+") OR (MH "Snow") OR (MH "Wildfires")
S2	TI (("natural disaster*" OR "natural catastrophe*" OR "natural hazard*" OR avalanche* OR landslide* OR mudslide* OR rockslide* OR earthquake* OR sinkhole* OR "volcanic eruption*" OR volcano* OR flood* OR tsunami* OR "tidal wave*" OR "ocean tide*" OR "earth tide*" OR hurricane* OR typhoon* OR "limnic eruption*" OR cyclone* OR "cyclonic storm*" OR blizzard* OR "ice storm*" OR "tropical storm*" OR hailstorm* OR "hail storm*" OR "cold wave*" OR "heat wave*" OR drought* OR thunderstorm* OR tornado* OR wildfire* OR "forest fire*" OR "wild fire*" OR "bush fire*" OR bushfire* OR "brush fire*" OR brushfire* OR "peat fire*" OR "vegetation fire*")) OR AB (("natural disaster*" OR "natural catastrophe*" OR "natural hazard*" OR avalanche* OR landslide* OR mudslide* OR rockslide* OR earthquake* OR sinkhole* OR "volcanic eruption*" OR volcano* OR flood* OR tsunami* OR "tidal wave*" OR "ocean tide*" OR "earth tide*" OR hurricane* OR typhoon* OR "limnic eruption*" OR cyclone* OR "cyclonic storm*" OR blizzard* OR "ice storm*" OR "tropical storm*" OR hailstorm* OR "hail storm*" OR "cold wave*" OR "heat wave*" OR drought* OR thunderstorm* OR tornado* OR wildfire* OR "forest fire*" OR "wild fire*" OR "bush fire*" OR bushfire* OR "brush fire*" OR brushfire* OR "peat fire*" OR "vegetation fire*"))
S3	S1 OR S2
S4	(MH "Embryo+") OR (MH "Fetus+") OR (MH "Expectant Mothers") OR (MH "Pregnancy") OR (MH "Maternal Exposure") OR (MH "Prenatal Exposure Delayed Effects")
S5	TI ((embryo* OR fetus* OR foetus* OR fetal* OR foetal* OR transplacenta* OR pregnan* OR trans-placenta* OR utero* OR intrauterine OR intra-uterine OR antenatal* OR ante-natal* OR antepartum OR ante-partum OR prenatal* OR pre-natal* OR intrapartum OR intra-partum OR perinatal* OR peri-natal* OR neonatal* OR neo-natal*)) OR AB ((embryo* OR fetus* OR foetus* OR fetal* OR foetal* OR transplacenta* OR pregnan* OR trans-placenta* OR utero* OR intrauterine OR intra-uterine OR antenatal* OR ante-natal* OR antepartum OR ante-partum OR prenatal* OR pre-natal* OR intrapartum OR intra-partum OR perinatal* OR peri-natal* OR neonatal* OR neo-natal*))
S6	S4 OR S5
S7	(MH "Child+") OR (MH "Adolescence+")

S8	TI ((baby OR babies OR neonate* OR neo-nate* OR newborn* OR new-born* OR infant* OR child* OR boy* OR girl* OR kid* OR pubescent* OR prepubescent* OR preteen* OR progen* OR juvenile* OR toddler* OR youth* OR offspring OR youngster* OR adolescen* OR teen*)) OR AB ((baby OR babies OR neonate* OR neo-nate* OR newborn* OR new-born* OR infant* OR child* OR boy* OR girl* OR kid* OR pubescent* OR prepubescent* OR preteen* OR progen* OR juvenile* OR toddler* OR youth* OR offspring OR youngster* OR adolescen* OR teen*))
S9	S7 OR S8
S10	S3 AND S6 AND S9
S11	S10 limit to (chinese or english or french)

Database	APA PsycNet (includes PsycInfo)
Line	Strategy
1	{Natural Disasters} OR {Temperature Effects}
2	(Title: "natural disaster*" OR Title: "natural catastrophe*" OR Title: "natural hazard*" OR Title: avalanche* OR Title: landslide* OR Title: mudslide* OR Title: rockslide* OR Title: earthquake* OR Title: sinkhole* OR Title: "volcanic eruption*" OR Title: volcano* OR Title: flood* OR Title: tsunami* OR Title: "tidal wave*" OR Title: "ocean tide*" OR Title: "earth tide*" OR Title: hurricane* OR Title: typhoon* OR Title: "limnic eruption*" OR Title: cyclone* OR Title: "cyclonic storm*" OR Title: blizzard* OR Title: "ice storm*" OR Title: "tropical storm*" OR Title: hailstorm* OR Title: "hail storm*" OR Title: "cold wave*" OR Title: "heat wave*" OR Title: drought* OR Title: thunderstorm* OR Title: tornado* OR Title: wildfire* OR Title: "forest fire*" OR Title: "wild fire*" OR Title: "bush fire*" OR Title: bushfire* OR Title: "brush fire*" OR Title: brushfire* OR Title: "peat fire*" OR Title: "vegetation fire*") OR (Abstract: "natural disaster*" OR Abstract: "natural catastrophe*" OR Abstract: "natural hazard*" OR Abstract: avalanche* OR Abstract: landslide* OR Abstract: mudslide* OR Abstract: rockslide* OR Abstract: earthquake* OR Abstract: sinkhole* OR Abstract: "volcanic eruption*" OR Abstract: volcano* OR Abstract: flood* OR Abstract: tsunami* OR Abstract: "tidal wave*" OR Abstract: "ocean tide*" OR Abstract: "earth tide*" OR Abstract: hurricane* OR Abstract: typhoon* OR Abstract: "limnic eruption*" OR Abstract: cyclone* OR Abstract: "cyclonic storm*" OR Abstract: blizzard* OR Abstract: "ice storm*" OR Abstract: "tropical storm*" OR Abstract: hailstorm* OR Abstract: "hail storm*" OR Abstract: "cold wave*" OR Abstract: "heat wave*" OR Abstract: drought* OR Abstract: thunderstorm* OR Abstract: tornado* OR Abstract: wildfire* OR Abstract: "forest fire*" OR Abstract: "wild fire*" OR Abstract: "bush fire*" OR Abstract: bushfire* OR Abstract: "brush fire*" OR Abstract: brushfire* OR Abstract: "peat fire*" OR Abstract: "vegetation fire*")
3	1 OR 2
4	{Prenatal Development} OR {Antepartum Period} OR {Prenatal Developmental Stages} OR {Embryo} OR {Fetus} OR {Prenatal Exposure} OR {Pregnancy} OR {Pregnancy Outcomes}
5	(Title: embryo* OR Title: fetus* OR Title: foetus* OR Title: fetal* OR Title: foetal* OR Title: transplacenta* OR Title: pregnan* OR Title: trans-placenta* OR Title: utero* OR Title: intrauterine OR Title: intra-uterine OR Title: antenatal* OR Title: ante-natal* OR Title: antepartum OR Title: ante-partum OR Title: prenatal* OR Title: pre-natal* OR Title: intrapartum OR Title: intra-partum OR Title: perinatal* OR Title: peri-natal* OR Title: neonatal* OR Title: neo-natal*) OR (Abstract: embryo* OR Abstract: fetus* OR Abstract: foetus* OR Abstract: fetal* OR Abstract: foetal* OR Abstract: transplacenta* OR Abstract: pregnan* OR Abstract: trans-placenta* OR Abstract: utero* OR Abstract: intrauterine OR Abstract: intra-uterine OR Abstract: antenatal* OR Abstract: ante-natal* OR Abstract: antepartum OR Abstract: ante-partum OR Abstract: prenatal* OR Abstract: pre-natal* OR Abstract: intrapartum OR Abstract: intra-partum OR Abstract: perinatal* OR Abstract: peri-natal* OR Abstract: neonatal* OR Abstract: neo-natal*)

6	4 OR 5
7	3 AND 6
8	7 with Childhood filter
9	7 with Adolescence filter
10	(Title: baby OR Title: babies OR Title: neonate* OR Title: neo-nate* OR Title: newborn* OR Title: new-born* OR Title: infant* OR Title: child* OR Title: boy* OR Title: girl* OR Title: kid* OR Title: pubescent* OR Title: prepubescent* OR Title: preteen* OR Title: progen* OR Title: juvenile* OR Title: toddler* OR Title: youth* OR Title: offspring OR Title: youngster* OR Title: adolescen* OR Title: teen*) OR (Abstract: baby OR Abstract: babies OR Abstract: neonate* OR Abstract: neo-nate* OR Abstract: newborn* OR Abstract: new-born* OR Abstract: infant* OR Abstract: child* OR Abstract: boy* OR Abstract: girl* OR Abstract: kid* OR Abstract: pubescent* OR Abstract: prepubescent* OR Abstract: preteen* OR Abstract: progen* OR Abstract: juvenile* OR Abstract: toddler* OR Abstract: youth* OR Abstract: offspring OR Abstract: youngster* OR Abstract: adolescen* OR Abstract: teen*)
11	7 AND 10
12	8 OR 9 OR 11

13	(((((((title: (embryo*)))) OR (((title: (fetus*)))) OR (((title: (foetus*)))) OR (((title: (fetal*)))) OR (((title: (foetal*)))) OR (((title: (transplacenta*)))) OR (((title: (pregnan*)))) OR (((title: (trans-placenta*)))) OR (((title: (utero*)))) OR (((title: (intrauterine)))) OR (((title: (intra-uterine)))) OR (((title: (antenatal*)))) OR (((title: (ante-natal*)))) OR (((title: (antepartum)))) OR (((title: (ante-partum)))) OR (((title: (prenatal*)))) OR (((title: (pre-natal*)))) OR (((title: (intrapartum)))) OR (((title: (intra-partum)))) OR (((title: (perinatal*)))) OR (((title: (peri-natal*)))) OR (((title: (neonatal*)))) OR (((title: (neo-natal*))))) OR ((((abstract: (embryo*)))) OR (((abstract: (fetus*)))) OR (((abstract: (foetus*)))) OR (((abstract: (fetal*)))) OR (((abstract: (foetal*)))) OR (((abstract: (transplacenta*)))) OR (((abstract: (pregnan*)))) OR (((abstract: (trans-placenta*)))) OR (((abstract: (utero*)))) OR (((abstract: (intrauterine)))) OR (((abstract: (intra-uterine)))) OR (((abstract: (antenatal*)))) OR (((abstract: (ante-natal*)))) OR (((abstract: (antepartum)))) OR (((abstract: (ante-partum)))) OR (((abstract: (prenatal*)))) OR (((abstract: (pre-natal*)))) OR (((abstract: (intrapartum)))) OR (((abstract: (intra-partum)))) OR (((abstract: (perinatal*)))) OR (((abstract: (peri-natal*)))) OR (((abstract: (neonatal*)))) OR (((abstract: (neo-natal*)))))) OR (((((IndexTermsFilt: ("Prenatal Development")))) OR (((IndexTermsFilt: ("Antepartum Period")))) OR (((IndexTermsFilt: ("Prenatal Developmental Stages")))) OR (((IndexTermsFilt: ("Embryo")))) OR (((IndexTermsFilt: ("Fetus")))) OR (((IndexTermsFilt: ("Prenatal Exposure")))) OR (((IndexTermsFilt: ("Pregnancy")))) OR (((IndexTermsFilt: ("Pregnancy Outcomes"))))))) AND ((((((title: ("natural disaster*")))) OR (((title: ("natural catastrophe*")))) OR (((title: ("natural hazard*")))) OR (((title: (avalanche*)))) OR (((title: (landslide*)))) OR (((title: (mudslide*)))) OR (((title: (rockslide*)))) OR (((title: (earthquake*)))) OR (((title: (sinkhole*)))) OR (((title: ("volcanic eruption*")))) OR (((title: (volcano*)))) OR (((title: (flood*)))) OR (((title: (tsunami*)))) OR (((title: ("tidal wave*")))) OR (((title: ("ocean tide*")))) OR (((title: ("earth tide*")))) OR (((title: (hurricane*)))) OR (((title: (typhoon*)))) OR (((title: ("limnic eruption*")))) OR (((title: (cyclone*)))) OR (((title: ("cyclonic storm*")))) OR (((title: (blizzard*)))) OR (((title: ("ice storm*")))) OR (((title: ("tropical storm*")))) OR (((title: (hailstorm*)))) OR (((title: ("hail storm*")))) OR (((title: ("cold wave*")))) OR (((title: ("heat wave*")))) OR (((title: (drought*)))) OR (((title: (thunderstorm*)))) OR (((title: (tornado*)))) OR (((title: (wildfire*)))) OR (((title: ("forest fire*")))) OR (((title: ("wild fire*")))) OR (((title: ("bush fire*")))) OR (((title: (bushfire*)))) OR (((title: ("brush fire*")))) OR (((title: (brushfire*)))) OR (((title: ("peat fire*")))) OR (((title: ("vegetation fire*"))))) OR ((((abstract: ("natural disaster*")))) OR (((abstract: ("natural catastrophe*")))) OR (((abstract: ("natural hazard*")))) OR (((abstract: (avalanche*)))) OR (((abstract: (landslide*)))) OR (((abstract: (mudslide*)))) OR (((abstract: (rockslide*)))) OR (((abstract: (earthquake*)))) OR (((abstract: (sinkhole*)))) OR (((abstract: ("volcanic eruption*")))) OR (((abstract: (volcano*)))) OR (((abstract: (flood*)))) OR (((abstract: (tsunami*)))) OR (((abstract: ("tidal wave*")))) OR (((abstract: ("ocean tide*")))) OR (((abstract: ("earth tide*")))) OR (((abstract: (hurricane*)))) OR (((abstract: (typhoon*)))) OR (((abstract: ("limnic eruption*")))) OR (((abstract: (cyclone*)))) OR (((abstract: ("cyclonic storm*")))) OR (((abstract: (blizzard*)))) OR (((abstract:

	("ice storm*")))) OR (((abstract: ("tropical storm*")))) OR (((abstract: (hailstorm*)))) OR (((abstract: ("hail storm*")))) OR (((abstract: ("cold wave*")))) OR (((abstract: ("heat wave*")))) OR (((abstract: (drought*)))) OR (((abstract: (thunderstorm*)))) OR (((abstract: (tornado*)))) OR (((abstract: (wildfire*)))) OR (((abstract: ("forest fire*")))) OR (((abstract: ("wild fire*")))) OR (((abstract: ("bush fire*")))) OR (((abstract: (bushfire*)))) OR (((abstract: ("brush fire*")))) OR (((abstract: (brushfire*)))) OR (((abstract: ("peat fire*")))) OR (((abstract: ("vegetation fire*")))))) OR (((((IndexTermsFilt: ("Natural Disasters")))) OR (((IndexTermsFilt: ("Temperature Effects")))))))) AND (((title: (baby)) OR (title: (babies)) OR (title: (neonate*)) OR (title: (neo-nate*)) OR (title: (newborn*)) OR (title: (new-born*)) OR (title: (infant*)) OR (title: (child*)) OR (title: (boy*)) OR (title: (girl*)) OR (title: (kid*)) OR (title: (pubescent*)) OR (title: (prepubescent*)) OR (title: (preteen*)) OR (title: (progen*)) OR (title: (juvenile*)) OR (title: (toddler*)) OR (title: (youth*)) OR (title: (offspring)) OR (title: (youngster*)) OR (title: (adolescen*)) OR (title: (teen*))) OR ((abstract: (baby)) OR (abstract: (babies)) OR (abstract: (neonate*)) OR (abstract: (neo-nate*)) OR (abstract: (newborn*)) OR (abstract: (new-born*)) OR (abstract: (infant*)) OR (abstract: (child*)) OR (abstract: (boy*)) OR (abstract: (girl*)) OR (abstract: (kid*)) OR (abstract: (pubescent*)) OR (abstract: (prepubescent*)) OR (abstract: (preteen*)) OR (abstract: (progen*)) OR (abstract: (juvenile*)) OR (abstract: (toddler*)) OR (abstract: (youth*)) OR (abstract: (offspring)) OR (abstract: (youngster*)) OR (abstract: (adolescen*)) OR (abstract: (teen*))))) OR ((((((title: (embryo*))) OR ((title: (fetus*))) OR ((title: (foetus*))) OR ((title: (fetal*))) OR ((title: (foetal*))) OR ((title: (transplacenta*))) OR ((title: (pregnan*))) OR ((title: (trans-placenta*))) OR ((title: (utero*))) OR ((title: (intrauterine))) OR ((title: (intra-uterine))) OR ((title: (antenatal*))) OR ((title: (ante-natal*))) OR ((title: (antepartum))) OR ((title: (ante-partum))) OR ((title: (prenatal*))) OR ((title: (pre-natal*))) OR ((title: (intrapartum))) OR ((title: (intra-partum))) OR ((title: (perinatal*))) OR ((title: (peri-natal*))) OR ((title: (neonatal*))) OR ((title: (neo-natal*)))) OR (((abstract: (embryo*))) OR ((abstract: (fetus*))) OR ((abstract: (foetus*))) OR ((abstract: (fetal*))) OR ((abstract: (foetal*))) OR ((abstract: (transplacenta*))) OR ((abstract: (pregnan*))) OR ((abstract: (trans-placenta*))) OR ((abstract: (utero*))) OR ((abstract: (intrauterine))) OR ((abstract: (intra-uterine))) OR ((abstract: (antenatal*))) OR ((abstract: (ante-natal*))) OR ((abstract: (antepartum))) OR ((abstract: (ante-partum))) OR ((abstract: (prenatal*))) OR ((abstract: (pre-natal*))) OR ((abstract: (intrapartum))) OR ((abstract: (intra-partum))) OR ((abstract: (perinatal*))) OR ((abstract: (peri-natal*))) OR ((abstract: (neonatal*))) OR ((abstract: (neo-natal*))))) OR ((((IndexTermsFilt: ("Prenatal Development"))) OR ((IndexTermsFilt: ("Antepartum Period"))) OR ((IndexTermsFilt: ("Prenatal Developmental Stages"))) OR ((IndexTermsFilt: ("Embryo"))) OR ((IndexTermsFilt: ("Fetus"))) OR ((IndexTermsFilt: ("Prenatal Exposure"))) OR ((IndexTermsFilt: ("Pregnancy"))) OR ((IndexTermsFilt: ("Pregnancy Outcomes")))))) AND (((((title: ("natural disaster*"))) OR ((title: ("natural catastrophe*"))) OR ((title: ("natural hazard*"))) OR ((title: (avalanche*))) OR ((title: (landslide*))) OR ((title: (mudslide*))) OR ((title: (rockslide*))) OR ((title: (earthquake*))) OR ((title: (sinkhole*))) OR ((title: ("volcanic eruption*"))) OR

	((title: (volcano*))) OR ((title: (flood*))) OR ((title: (tsunami*))) OR ((title: ("tidal wave*"))) OR ((title: ("ocean tide*"))) OR ((title: ("earth tide*"))) OR ((title: (hurricane*))) OR ((title: (typhoon*))) OR ((title: ("limnic eruption*"))) OR ((title: (cyclone*))) OR ((title: ("cyclonic storm*"))) OR ((title: (blizzard*))) OR ((title: ("ice storm*"))) OR ((title: ("tropical storm*"))) OR ((title: (hailstorm*))) OR ((title: ("hail storm*"))) OR ((title: ("cold wave*"))) OR ((title: ("heat wave*"))) OR ((title: (drought*))) OR ((title: (thunderstorm*))) OR ((title: (tornado*))) OR ((title: (wildfire*))) OR ((title: ("forest fire*"))) OR ((title: ("wild fire*"))) OR ((title: ("bush fire*"))) OR ((title: (bushfire*))) OR ((title: ("brush fire*"))) OR ((title: (brushfire*))) OR ((title: ("peat fire*"))) OR ((title: ("vegetation fire*")))) OR (((abstract: ("natural disaster*"))) OR ((abstract: ("natural catastrophe*"))) OR ((abstract: ("natural hazard*"))) OR ((abstract: (avalanche*))) OR ((abstract: (landslide*))) OR ((abstract: (mudslide*))) OR ((abstract: (rockslide*))) OR ((abstract: (earthquake*))) OR ((abstract: (sinkhole*))) OR ((abstract: ("volcanic eruption*"))) OR ((abstract: (volcano*))) OR ((abstract: (flood*))) OR ((abstract: (tsunami*))) OR ((abstract: ("tidal wave*"))) OR ((abstract: ("ocean tide*"))) OR ((abstract: ("earth tide*"))) OR ((abstract: (hurricane*))) OR ((abstract: (typhoon*))) OR ((abstract: ("limnic eruption*"))) OR ((abstract: (cyclone*))) OR ((abstract: ("cyclonic storm*"))) OR ((abstract: (blizzard*))) OR ((abstract: ("ice storm*"))) OR ((abstract: ("tropical storm*"))) OR ((abstract: (hailstorm*))) OR ((abstract: ("hail storm*"))) OR ((abstract: ("cold wave*"))) OR ((abstract: ("heat wave*"))) OR ((abstract: (drought*))) OR ((abstract: (thunderstorm*))) OR ((abstract: (tornado*))) OR ((abstract: (wildfire*))) OR ((abstract: ("forest fire*"))) OR ((abstract: ("wild fire*"))) OR ((abstract: ("bush fire*"))) OR ((abstract: (bushfire*))) OR ((abstract: ("brush fire*"))) OR ((abstract: (brushfire*))) OR ((abstract: ("peat fire*"))) OR ((abstract: ("vegetation fire*"))))) OR ((((IndexTermsFilt: ("Natural Disasters"))) OR ((IndexTermsFilt: ("Temperature Effects"))))))) AND ((AgeGroupFilt: "Childhood (birth-12 yrs)"))) OR ((((((title: (embryo*))) OR ((title: (fetus*))) OR ((title: (foetus*))) OR ((title: (fetal*))) OR ((title: (foetal*))) OR ((title: (transplacenta*))) OR ((title: (pregnan*))) OR ((title: (trans-placenta*))) OR ((title: (utero*))) OR ((title: (intrauterine))) OR ((title: (intra-uterine))) OR ((title: (antenatal*))) OR ((title: (ante-natal*))) OR ((title: (antepartum))) OR ((title: (ante-partum))) OR ((title: (prenatal*))) OR ((title: (pre-natal*))) OR ((title: (intrapartum))) OR ((title: (intra-partum))) OR ((title: (perinatal*))) OR ((title: (peri-natal*))) OR ((title: (neonatal*))) OR ((title: (neo-natal*)))) OR (((abstract: (embryo*))) OR ((abstract: (fetus*))) OR ((abstract: (foetus*))) OR ((abstract: (fetal*))) OR ((abstract: (foetal*))) OR ((abstract: (transplacenta*))) OR ((abstract: (pregnan*))) OR ((abstract: (trans-placenta*))) OR ((abstract: (utero*))) OR ((abstract: (intrauterine))) OR ((abstract: (intra-uterine))) OR ((abstract: (antenatal*))) OR ((abstract: (ante-natal*))) OR ((abstract: (antepartum))) OR ((abstract: (ante-partum))) OR ((abstract: (prenatal*))) OR ((abstract: (pre-natal*))) OR ((abstract: (intrapartum))) OR ((abstract: (intra-partum))) OR ((abstract: (perinatal*))) OR ((abstract: (peri-natal*))) OR ((abstract: (neonatal*))) OR ((abstract: (neo-natal*))))) OR ((((IndexTermsFilt: ("Prenatal Development"))) OR ((IndexTermsFilt: ("Antepartum Period"))) OR ((IndexTermsFilt: ("Prenatal Developmental Stages"))) OR ((IndexTermsFilt:

	("Embryo"))) OR ((IndexTermsFilt: ("Fetus"))) OR ((IndexTermsFilt: ("Prenatal Exposure"))) OR ((IndexTermsFilt: ("Pregnancy"))) OR ((IndexTermsFilt: ("Pregnancy Outcomes")))))) AND (((((title: ("natural disaster*"))) OR ((title: ("natural catastrophe*"))) OR ((title: ("natural hazard*"))) OR ((title: (avalanche*))) OR ((title: (landslide*))) OR ((title: (mudslide*))) OR ((title: (rockslide*))) OR ((title: (earthquake*))) OR ((title: (sinkhole*))) OR ((title: ("volcanic eruption*"))) OR ((title: (volcano*))) OR ((title: (flood*))) OR ((title: (tsunami*))) OR ((title: ("tidal wave*"))) OR ((title: ("ocean tide*"))) OR ((title: ("earth tide*"))) OR ((title: (hurricane*))) OR ((title: (typhoon*))) OR ((title: ("limnic eruption*"))) OR ((title: (cyclone*))) OR ((title: ("cyclonic storm*"))) OR ((title: (blizzard*))) OR ((title: ("ice storm*"))) OR ((title: ("tropical storm*"))) OR ((title: (hailstorm*))) OR ((title: ("hail storm*"))) OR ((title: ("cold wave*"))) OR ((title: ("heat wave*"))) OR ((title: (drought*))) OR ((title: (thunderstorm*))) OR ((title: (tornado*))) OR ((title: (wildfire*))) OR ((title: ("forest fire*"))) OR ((title: ("wild fire*"))) OR ((title: ("bush fire*"))) OR ((title: (bushfire*))) OR ((title: ("brush fire*"))) OR ((title: (brushfire*))) OR ((title: ("peat fire*"))) OR ((title: ("vegetation fire*")))) OR (((abstract: ("natural disaster*"))) OR ((abstract: ("natural catastrophe*"))) OR ((abstract: ("natural hazard*"))) OR ((abstract: (avalanche*))) OR ((abstract: (landslide*))) OR ((abstract: (mudslide*))) OR ((abstract: (rockslide*))) OR ((abstract: (earthquake*))) OR ((abstract: (sinkhole*))) OR ((abstract: ("volcanic eruption*"))) OR ((abstract: (volcano*))) OR ((abstract: (flood*))) OR ((abstract: (tsunami*))) OR ((abstract: ("tidal wave*"))) OR ((abstract: ("ocean tide*"))) OR ((abstract: ("earth tide*"))) OR ((abstract: (hurricane*))) OR ((abstract: (typhoon*))) OR ((abstract: ("limnic eruption*"))) OR ((abstract: (cyclone*))) OR ((abstract: ("cyclonic storm*"))) OR ((abstract: (blizzard*))) OR ((abstract: ("ice storm*"))) OR ((abstract: ("tropical storm*"))) OR ((abstract: (hailstorm*))) OR ((abstract: ("hail storm*"))) OR ((abstract: ("cold wave*"))) OR ((abstract: ("heat wave*"))) OR ((abstract: (drought*))) OR ((abstract: (thunderstorm*))) OR ((abstract: (tornado*))) OR ((abstract: (wildfire*))) OR ((abstract: ("forest fire*"))) OR ((abstract: ("wild fire*"))) OR ((abstract: ("bush fire*"))) OR ((abstract: (bushfire*))) OR ((abstract: ("brush fire*"))) OR ((abstract: (brushfire*))) OR ((abstract: ("peat fire*"))) OR ((abstract: ("vegetation fire*"))))) OR ((((IndexTermsFilt: ("Natural Disasters"))) OR ((IndexTermsFilt: ("Temperature Effects"))))))) AND ((AgeGroupFilt: "Adolescence (13-17 yrs)")))
14	limit 13 to humans
